# Dissecting the Transcriptional and Chromatin Accessibility Heterogeneity of Proliferating Cone Precursors in Human Retinoblastoma Tumors by Single Cell Sequencing—Opening Pathways to New Therapeutic Strategies?

**DOI:** 10.1167/iovs.62.6.18

**Published:** 2021-05-17

**Authors:** Joseph Collin, Rachel Queen, Darin Zerti, David H Steel, Claire Bowen, Manoj Parulekar, Majlinda Lako

**Affiliations:** 1 Biosciences Institute, Newcastle University, Newcastle, United Kingdom; 2 Birmingham Women's and Children NHS Foundation Trust, Birmingham, United Kingdom

**Keywords:** retinoblastoma, tumor, scRNA-Seq, scATAC-Seq, proliferating cones, cell of origin

## Abstract

**Purpose:**

Retinoblastoma (Rb) is a malignant neoplasm arising during retinal development from mutations in the RB1 gene. Loss or inactivation of both copies of RB1 results in initiation of retinoblastoma tumors; however, additional genetic changes are needed for the continued growth and spread of the tumor. Ex vivo research has shown that in humans, retinoblastoma may initiate from RB1-depleted cone precursors. Notwithstanding, it has not been possible to assess the full spectrum of clonal types within the tumor itself in vivo and the molecular changes occurring at the cells of origin, enabling their malignant conversion. To overcome these challenges, we have performed the first single cell (sc) RNA- and ATAC-Seq analyses of primary tumor tissues, enabling us to dissect the transcriptional and chromatin accessibility heterogeneity of proliferating cone precursors in human Rb tumors.

**Methods:**

Two Rb tumors each characterized by two pathogenic *RB1* mutations were dissociated to single cells and subjected to scRNA-Seq and scATAC-Seq using the 10× Genomics platform. In addition, nine human embryonic and fetal retina samples were dissociated to single cells and subjected to scRNA- and ATAC-Seq analyses. The scRNA- and ATAC-Seq data were embedded using Uniform Manifold Approximation and Projection and clustered with Seurat graph–based clustering. Integrated scATAC-Seq analysis of Rb tumors and human embryonic/fetal retina samples was performed to identify Rb cone enriched subclusters. Pseudo time analysis of proliferating cones in the Rb samples was performed with Monocle. Ingenuity Pathway Analysis was used to identify the signaling pathway and upstream regulators in the Rb cone–enriched subclusters.

**Results:**

Our single cell analyses revealed the predominant presence of cone precursors at different stages of the cell cycle in the Rb tumors and among those identified the G2/M subset as the cell type of origin. scATAC-Seq analysis identified two Rb enriched cone subclusters, each characterized by activation of different upstream regulators and signaling pathways, enabling proliferating cone precursors to escape cell cycle arrest and/or apoptosis.

**Conclusions:**

Our study provides evidence of Rb tumor heterogeneity and defines molecular pathways that can be targeted to define new treatment strategies.

Retinoblastoma (Rb) is a childhood cancer of the developing retina caused by biallelic inactivation of *RB1* or *MYCN* amplification in a susceptible retinal cell type.^1^ The frequency of Rb is estimated at about one in 15,000 live births. It accounts for 7% to 17% of all tumors (4% to 29% of solid tumors) in infancy and around 4% to 6% of all cancers in children younger than 15 years old.^2^^–^^4^ Globally 8600 to 9000 children are affected each year, with 40 to 50 new cases being diagnosed in the UK alone.^5^ Approximately 40% of all cases are bilateral (germline), and 60% are unilateral (of which 15% are germline and the rest somatic). Most Rb cases are diagnosed below the age of 5 years. Treatment, which depends on the number, position and size of the tumors in the eye, leads to a good survival rate (85%–97% for unilateral cases and 88%–100% for bilateral cases) but can result in significant vision deficits and adversely affect quality of life.^5^

Rb tumors have a propensity for the central retina and appear in a centrifugal fashion as the child grows. This correlates with the distribution of cones, which are concentrated centrally and mature in a centrifugal fashion.

Knudson^6^ proposed that two separate mutational events (M_1_ and M_2_), which result in loss or inactivation of both copies of the *RB1* gene, are required to initiate the tumor. Recent whole genome sequencing studies have shown that the genome of Rb cells is more stable than previously believed and that few genetic changes are required for Rb development after *RB1* inactivation. Instead, epigenetic deregulation of tumor-promoting pathways cooperating with the loss of RB1 has been shown to be required for tumorigenesis.^7^

Recent research has shown that in humans, Rb tumors may initiate from RB1-depleted cone precursors that are in a particular maturation stage and are able to form premalignant retinoma before retinoblastoma lesions.^8^ However, molecular and cellular analysis indicates that induced Rb tumors in mice have properties of amacrine/horizontal interneurons, suggesting a different tumor cell of origin.^9^ In addition, inactivation of one copy of *Rb1* does not lead to the development of murine retinoblastoma at a detectable frequency. Furthermore, no eye abnormalities are observed on inactivation of both copies of *Rb1.*^10^^–^^12^ This indicates that different mechanisms underlying Rb exist between human and mice.

The cell of origin studies in human Rb have been conducted using RNA interference to knock down *RB1* gene, followed by assessment of malignant conversion of cells in vitro or through the xenograft model in vivo*,*^8^^,^^13^ yielding important insights into gene expression and epigenetic differences between human and mouse retinoblastoma tumors. In parallel, pluripotent stem cell derived organoid retinoblastoma models have recently been generated, corroborating the Arrestin 3^+^ maturing cone precursors as cell of origin for Rb.^14^ Next-generation sequencing methods have enabled detection of mutations in peripheral blood samples at a low level of mosaicism that would have been missed with the traditional sequencing methods. Notwithstanding, it has not been possible to assess the full spectrum of clonal types within the tumor or the molecular changes occurring at the cells of origin, which are likely to be characterized by a different transcriptional and epigenetic profile. Recent single cell (sc) sequencing technologies, combined with multisampling of human tumors, have yielded important insights into tumor heterogeneity, dissecting the tumor microenvironment and molecular pathways involved in malignant conversion, which can be exploited for effective cancer treatments.^15^ In this article, we describe the first application of scRNA- and ATAC-Seq analyses to Rb tumors, showing the predominant presence of cycling cone precursors and identifying the G2/M cones as the cell type of origin. Our single cell analyses reveal the presence of two Rb cone–enriched subclusters associated with activation of different molecular pathways and upstream regulators, which lead to resistance to cell cycle arrest or apoptosis.

## Methods

### ScRNA- and -ATAC-Seq

Nine samples of developing human eyes from 10 to 21 postconception weeks were obtained from the Human Developmental Biology Resource under ethics permission 08/H0906/21+5 issued by the North East-Newcastle and North Tyneside 1 Research Ethics Committee. Two Rb samples were obtained from the Children with Cancer and Leukaemia group Tissue Bank after informed consent was obtained by the parents under ethics permission 18/EM/0134 issued by the East Midlands-Derby Research Ethics Committee. The samples were obtained after cutting open the freshly enucleated eyes. Because the posterior segment of the eyes was full of tumor, a small section of sclera was removed, and then the small tumor samples were obtained with a curette under direct visualization. Both samples were from markedly dedifferentiated tumors. One of the eyes had postlaminar optic nerve invasion. Human developmental retina samples were isolated and dissociated to single cells using a neurosphere dissociation kit (Miltenyi Biotech, Auburn, CA, USA). The same dissociation procedure was performed for the Rb tumors. 10,000 cells from each sample were captured, and sequencing libraries generated using the Chromium Single Cell 3′ Library & Gel Bead Kit (version 3; 10x Genomics, Pleasanton, CA, USA). For nine developing retinas and two retinoblastoma samples, a nuclei preparation from the remaining dissociated cells was performed. Ten thousand of the subsequent nuclei were captured, and sequencing libraries generated using the Chromium Single Cell ATAC Library & Gel Bead Kit (version 1, 10x Genomics). Single cell RNA-Seq libraries were sequenced to 50,000 reads per cell and scATAC-Seq libraries were sequenced to 25,000 reads per nucleus on an Illumina NovaSeq 6000 (Illumina, San Diego, CA, USA).

### ScRNA-Seq Analysis

CellRanger version 3.01 (10x Genomics) was used to de-multiplex the samples and align and quantify them against the human reference genome GRCh38. Cells were filtered where fewer than 1000 reads or 500 genes or greater than 15% mitochondrial reads. Diagnostic plots were used to determine the most appropriate thresholds for our data. DoubletFinder was used to predict doublets in the data which were then filtered.

In total, 8086 cells from the 2 Rb tumors passed the quality control and filtering steps and were used for downstream analysis. The Seurat R package (version 3.1.3; Satija Lab, New York, NY, USA) was used to normalize individual experiments using the “LogNormalize” method. The two Rb datasets were combined using the Seurat standard integrated analysis approach, which was used to overcome batch effects and donor differences.^16^ A subset of 2000 highly variable genes and the first 10 principle components were used for integration. A combined dataset was created by finding anchors between cells in the individual datasets to create a batch corrected expression matrix. The Seurat graph–based method was used to cluster the data, and a resolution of 1 was chosen, which produced 18 clusters. Seurat was used to predict the cell cycle state for each cell in the dataset. The clustering results and cell cycle predictions were visualized using uniform manifold approximation and projection.

The FindMarkers function from Seurat was used to identify markers genes within each cluster. Cell types were then assigned to these clusters and annotated using these genes lists. Clusters were annotated using a panel of known retinal cell type specific marker genes (Supplementary Table S1).

Cells from clusters 0 to 9, 11, 12, and 14, which were identified as cone cells, were selected from the datasets. Monocle 3 was used to order the cells and infer a pseudo time trajectory within the selected cells.

### scATAC-ATAC Analysis

Peaks were detected using Cellranger ATAC software (version 1.2) in each of the samples. A set of shared peaks was then defined using the Cellranger ATAC aggregate function. The combined datasets were imported using Signac, and quality control steps were performed to remove cells that did not meet the following thresholds: at least 50,000 reads in peaks, more than 50% of reads in fragments, less than 0.05% of reads in Ensembl Blacklist, a nucleosome signal of less than 10, and a transcription start sites (TSS) enrichment >2. A gene activity matrix was calculated in the ATAC-Seq dataset, using the genes closest to the peaks.

The samples were grouped by stage or disease into the following groups: 10 postconception weeks (PCW), 12PCW, 15-16PCW, 20-21PCW, and Rb, and datasets were down sampled to 5000 cells for each group. Signac was used to identify a set of features, and then a latent semantic indexing dimension was applied to the data. Harmony was used to correct for batch differences. The cells were then clustered at resolution of 1 using Seurat and embedded using Uniform Manifold Approximation and Projection. Differential accessibility analysis was used to test for significant differences in open regions between clusters. Clusters were annotated by linking differential accessible peaks to the nearest genes. Peaks were linked to promoters where they overlapped and distal or intergenic regions of any transcription start site (−1000bp, +100bp). Cluster 4 and cluster 15 were identified as cone clusters. The two cone clusters and Rb samples were then selected, and subclustering was performed as described above.

Peaks were linked to promoters using annotation from the Cellranger ATAC pipeline. The JEME database^17^ was used to predict peaks in enhancer regions, and the JASPAR 2020 database^18^ was used to look for the presence of motifs in differentially accessible peaks.

The differentially accessible peaks were analyzed using Qiagen Ingenuity Pathway Analysis (IPA). The lists of promoters were used to predict upstream regulators, and motif data were overlaid onto the prediction to look for consensus in the predictions.

### Ocular Histopathology

After enucleation, eyes were fixed in 4% paraformaldehyde overnight at 4°C, dehydrated through an alcohol series, and washed in xylene. The samples were then embedded in paraffin, and 5-µm sagittal sections were cut through the optic nerve for histopathological analysis.^7^ The tissue sections were deparaffinized in xylene and rehydrated through several incubations of graded ethanol washes followed by two washing steps in PBS. Finally, the slides were counterstained by hematoxylin stain followed by light microscopic examination for ocular histopathological parameter assessment such as tumor differentiation and invasion, evaluation of the involvement of the optic nerve, and presence or absence of the Flexner-Wintersteiner rosettes.

## Results

### scRNA-Seq Analysis Identifies G2/M Cone Precursors as Cell of Origin For Retinoblastoma Tumors

**Figure 1.**
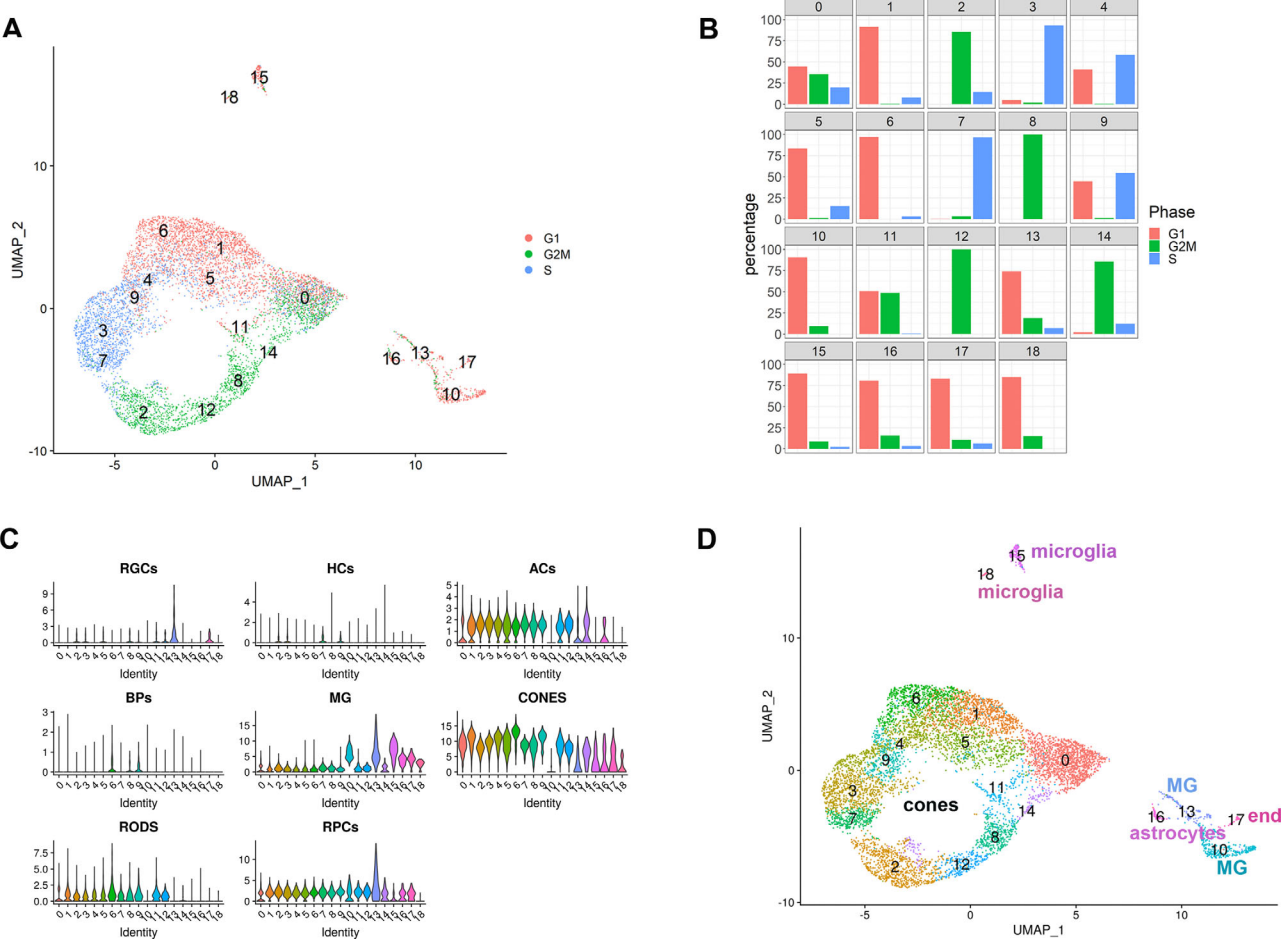
Dominant presence of cone precursors in Rb tumors revealed by scRNA-Seq analysis. (**A**) Integrated Uniform Manifold Approximation and Projection (UMAP) of human Rb tumors showing the presence of 18 cell clusters; (**B**) Cell cycle profile of 18 cell clusters; (**C**) Violin plots showing expression of retinal cell type specific markers in the 18 cell clusters; (**D**) Annotated UMAP showing the cell cluster identity. ACs, amacrine cells; BPs, bipolar cells; HCs, horizontal cells; MG, Muller Glia cells; RGCs, retinal ganglion cells.

Two retinoblastoma tumor samples were obtained from a 4.5-month-old male child diagnosed with aggressive early presentation left unilateral non-germline retinoblastoma and a 34-month-old male child diagnosed with left eye group E germline retinoblastoma at presentation (Supplementary Figs. S1, S2). Two pathogenic *RB1* mutations were identified in each of the tumor samples (Table). After eye enucleation and tumor retrieval, single cell dissociation and cell capture, scRNA-Seq was carried out. Clustering analysis of the 8,086 cells from both donors, which passed quality control thresholds, revealed the presence of 18 cell clusters (Fig. 1A).

**Table.** Summary of Clinical Information and RB1 Pathogenic Mutations of the Rb Patients

Patient	Age	Sex	Genetics	Clinical Information
Retinoblastoma 1	4 months	Male	*RB1 c.409G > T* p. (Glu137Ter) – Pathogenic *RB1 c.1965T > G* p.(Tyr655Ter) – Pathogenic	Affected Eye: Enlarged corneal diameter with raised intraocular pressure of 38 mmHg (with I-care tonometry whilst awake). Corneal edema with very shallow anterior chamber, peripheral anterior synechiae and altered iris pattern. Retinal vessels visible up against the posterior surface of the lens, and mass visualized appearing to extend to the ora serrata. Ultrasound B scan confirms the presence of a mass occupying the entire posterior segment of the left eye, with multiple areas of calcification, consistent with presence of retinoblastoma. Enucleation was performed. Pathology confirmed the presence of exophytic tumor, focal necrosis and dystrophic calcification and small round hyperchromatic cells arranged into rosettes.
Retinoblastoma 2	34 months	Male	*RB1 exon 3 to exon 6 deletion* – Pathogenic *RB1 c.763C > T* p. (Arg255Ter) – Pathogenic	Affected Eye: Presence of inferotemporal mass with calcification and active vitreous seeds pre-retinally along the ora serrata for 360 degrees, and a “pseudo-hypopyon” at the posterior pole. The anterior edge of the tumor is not fundoscopically identifiable with indentation. High frequency ultrasound biomicroscopy revealed seeding onto the anterior zonules in the posterior chamber and seeding onto the anterior crystalline lens behind the iris and suspended free-floating clumps of cells, which were identifiable optically and ultrasonographically in the anterior chamber. Gonioscopy was normal and the intraocular pressure was not raised. Subtle iris hypochromia was noted. Enucleation was performed.

Cell cycle analysis identified a large number of cycling clusters (9 out of 18), containing more than 45% of cells in S- (clusters 3, 4, 7, and 9) or G2/M- phase of the cell cycle (clusters 2, 8, 11, 12, and 14) (Fig. 1B). In accordance, expression of cell cycle regulators involved in S and G2/M phase progression (*CCNE1, CCNE2, CCNB2, CCNA2, CDK1*) and proliferation markers (*MKi67, PCNA*) was highest in these cycling cell clusters (Supplementary Fig. S3). Expression of *RB1* was very low in most clusters (2–12 and 15) and absent from some others (Supplementary Fig. S3), and moreover it did not correlate with the clusters cell cycle distribution profiles (Fig. 1B). Assessment of all four *RB1*-detected mutations in the two Rb tumor samples by Mutation Taster program indicated that the mutant *RB1* transcripts were most likely to undergo nonsense-mediated decay, resulting in loss of RB1 protein. Expression of RB1-related family members *RBL1* and *RBL2* was low and restricted to fewer cell clusters compared to *RB1*. In contrast, expression of *E2F1*, the transcription factor that binds RB1 in a cell type–specific manner and regulates G1 to S cell cycle progression, was highest in S phase clusters, less so in G2/M clusters, and absent in clusters 10 and 15 to 18 (Supplementary Fig. S3). *E2F2* and *E2F3* showed a lower and more restricted cluster expression compared to *E2F1*.

Activation of E2F1 has been shown to lead to an increase in p14 (CDKN2A) expression, which is thought to stabilize p53, leading to cell-cycle arrest or apoptosis, unless a second lesion (such as a mutation in *p14^ARF^* or *p53* itself^19^) occurs. Our data corroborate these previous published findings, showing prominent expression of p14 in the same cell clusters as E2F1 (Supplementary Fig. S3). We also investigated the expression of two critical mediators of pRB-induced cell cycle arrest, p27 (CDKN1B) and SKP2. In the presence of growth promoting signals, Skp2 binds p27, resulting in its degradation; however, in the absence of such signals, pRB binds Skp2, resulting in its degradation, p27 accumulation and G1 arrest. Our scRNA-Seq data show low *SKP2* expression in 6/9 cycling clusters; contrary to expected p27 degradation occurring when pRB is lacking, we observed expression of p27 in all cycling clusters (Supplementary Fig. S3), which suggests a dysfunctional SKP2-p27 regulation that may enable these cells to bypass G1 arrest.

We then performed cluster identification using marker gene expression (Supplementary Table S2); however, the high expression of cell cycle–related genes in cycling clusters masked expression of cell type–specific markers. Hence, violin plots with key markers of each retinal cell lineage identified in recent scRNA and bulk RNA-Seq studies^20^^,^^21^ of developing and adult human retina were generated. This analysis showed high and predominant expression of cone markers in clusters 0 to 9, 11, 12, and 14 (Figs. 1C and 1D), microglia in clusters 15 and 18, astrocytes in cluster 16, Muller glia in clusters 10 and 13 and endothelial markers in cluster 17. Detailed ridge-plots showed high expression of cone Arrestin 3 (*ARR3*) in the predicted cone clusters 0-9, 11, 12 and 14 (Supplementary Fig. S4). Cone Arrestin 3 has been shown to be expressed in human cone precursors that enter the cell cycle in retinoblastoma.^8^ Furthermore, the Rb cone clusters organized in an apparent cyclic form dictated by the dynamic cell cycle data, expressed other cone precursor markers at a high level including *RXRG, THRB,* as well as genes involved in the visual cycle (*GNAT2, GNB3, PDE6H*). The expression of rod precursor markers was variable, with *NRL* expressed in all cone clusters and *NR2E3* absent. The expression of mature cone markers was absent (Supplementary Fig. S4), indicating that cone clusters 0 to 9, 11, 12, and 14 represent *cone precursors* at different phases of the cell cycle.

MDM2 and MYCN oncoproteins are highly expressed during human cone maturation. Their expression together with those of RXRG and THRB2 is essential for the proliferation of Rb-depleted cone precursors.^22^^,^^23^ In accordance, *MDM2* and *MYCN* expression was observed in all the cone clusters, except cluster 0, where the expression of both genes was absent (Supplementary Fig. S5). *MYC* was also expressed, but at the lower level than *MYCN*, whereas *MDM4* was expressed in all the cone clusters at higher levels compared to *MDM2*. Although mutations in *RB1* are often considered as the key prerequisite for retinoblastoma initiation, further genetic changes that may activate oncogenes or inactivate tumor suppressor genes may drive the acquisition of the malignant phenotype.^24^ Genetic studies have shown copy number changes in key genes such as *KLF4, DDX1, MDM4, OTX2* and loss in *DEK, CDH1, BCOR* and *RBL2*. The scRNA-Seq analysis shows *OTX2, MDM4, DDX1* expression in most of the cone populations (Supplementary Fig. S5) and no expression of *CDH1*, corroborating these published data. The tumor cone precursors were also characterized by the expression of *DEK* and *BCOR*, despite suggested loss of copy of number (Supplementary Fig. S5). Furthermore, expression of *BRCA1* and *BRCA2*, both required for embryonic cellular proliferation in mouse^25^ was also observed in most cone clusters but not all.

Given the highly proliferative nature of majority of cone clusters identified in the Rb tumors, we went on to investigate the expression of retinal progenitor cell (RPCs) markers. Although SIX3 and SIX6 are jointly required to maintain the multipotent RPCs, we only observed high expression of *SIX6* in the tumor cone clusters (Supplementary Fig. S6).^26^ This together with lack of expression of other well-known RPCs markers such as *SOX2, SFRP2, HES1* and *HES5*, excludes RPCs as cell of origin for retinoblastoma. *SOX4* and *SOX11*, markers of neurogenic RPCs, were also expressed in most tumor cone clusters. Overexpression of both genes in murine RPCs results in increased cones at the expense of rods and Muller glia cells.^27^ Moreover, *Sox11* overexpression has been shown to lead to cone genesis even after the normal cone genesis window; hence, their expression in the tumor cone clusters may suggest their involvement in the underlying cone-rich tumor phenotype in Rb.

**Figure 2.**
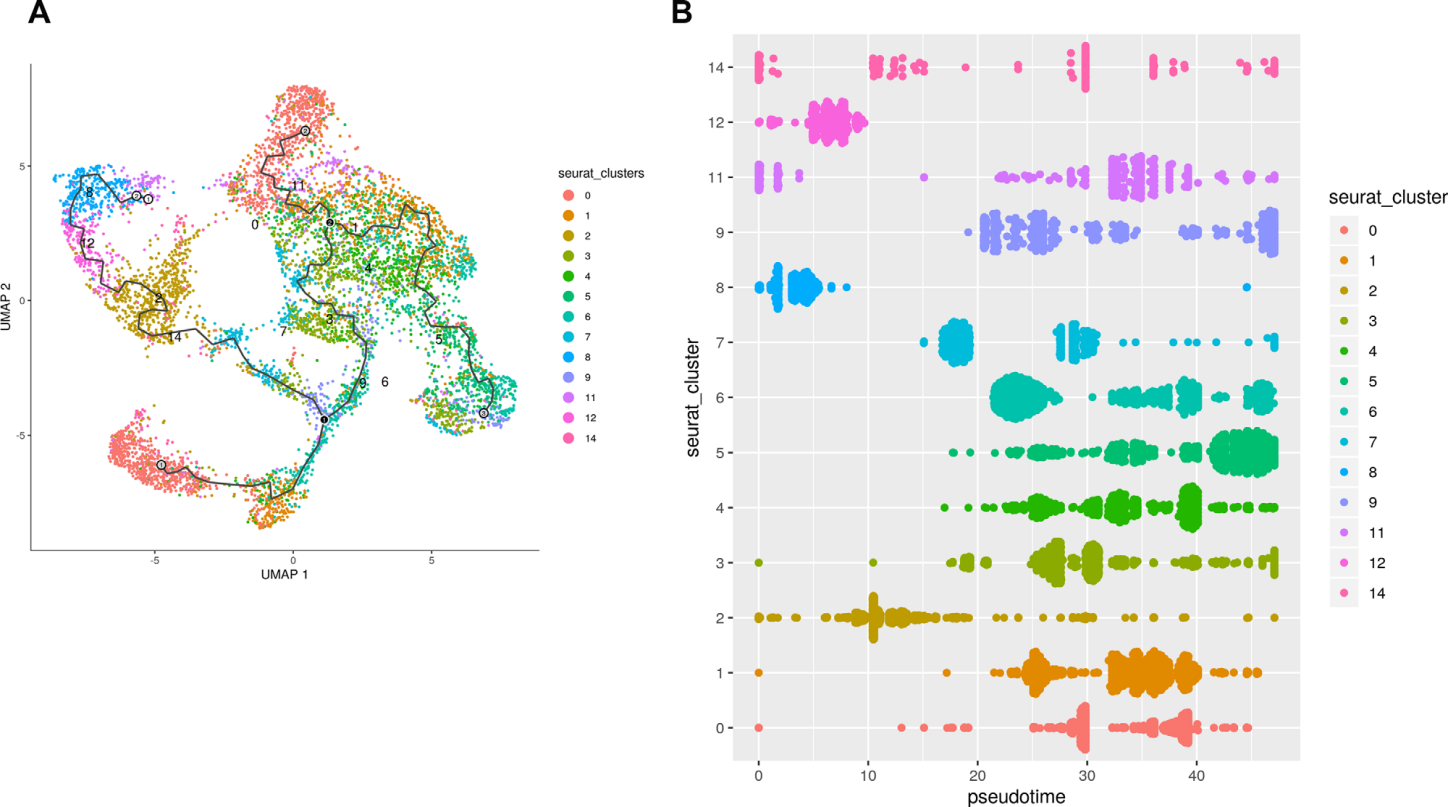
Pseudo time analysis reveals the G2/M cone precursors as the cell of origin for Rb tumors. (**A**) Pseudo time analysis of Rb cone clusters; (**B**) Order of cell emergence shown by cone cluster type.

Pseudo time analysis of all tumor cone clusters was carried out with Monocle. The G2/M clusters 8, 12, 2, and 14 were positioned at the start of the tree, followed by the clusters predominantly in S phase (7, 9, 3, and 4) and the clusters predominantly in G1 (0, 1, 6, and 5) (Figs. 2A, 2B). Together these data point to G2/M cone precursors as cell of origin for retinoblastoma, corroborating published findings of human retinoblastoma tumors arising from Rb-deficient cone precursors.^22^

### ScATAC-Seq Analysis Reveals the Presence of Two Rb Tumor Specific Enriched Cone Subclusters

Work performed by Xu and colleagues^22^ suggest that *proliferation of cone precursors* in the absence of programmed cell death during retinal development is the key cellular event that leads to the formation of Rb tumors. To gain insights into the molecular events that lead to cone proliferation during human retinal development, we performed scRNA-Seq and scATAC-Seq analyses of 9 embryonic and fetal retina samples spanning 10PCW to 21PCW and the two retinoblastoma tumors described above. Given the dominant influence of cell cycle events in the scRNA-Seq analysis, we opted to perform an integrated scATAC-Seq analysis, which clusters cells on the basis of chromatin accessibility and is thus more likely to identify early epigenetic and transcriptional events that lead to cone precursor proliferation and unravel different clones within the tumor that are likely to arise as result of independent second lesion in addition to *RB1* inactivation.

**Figure 3.**
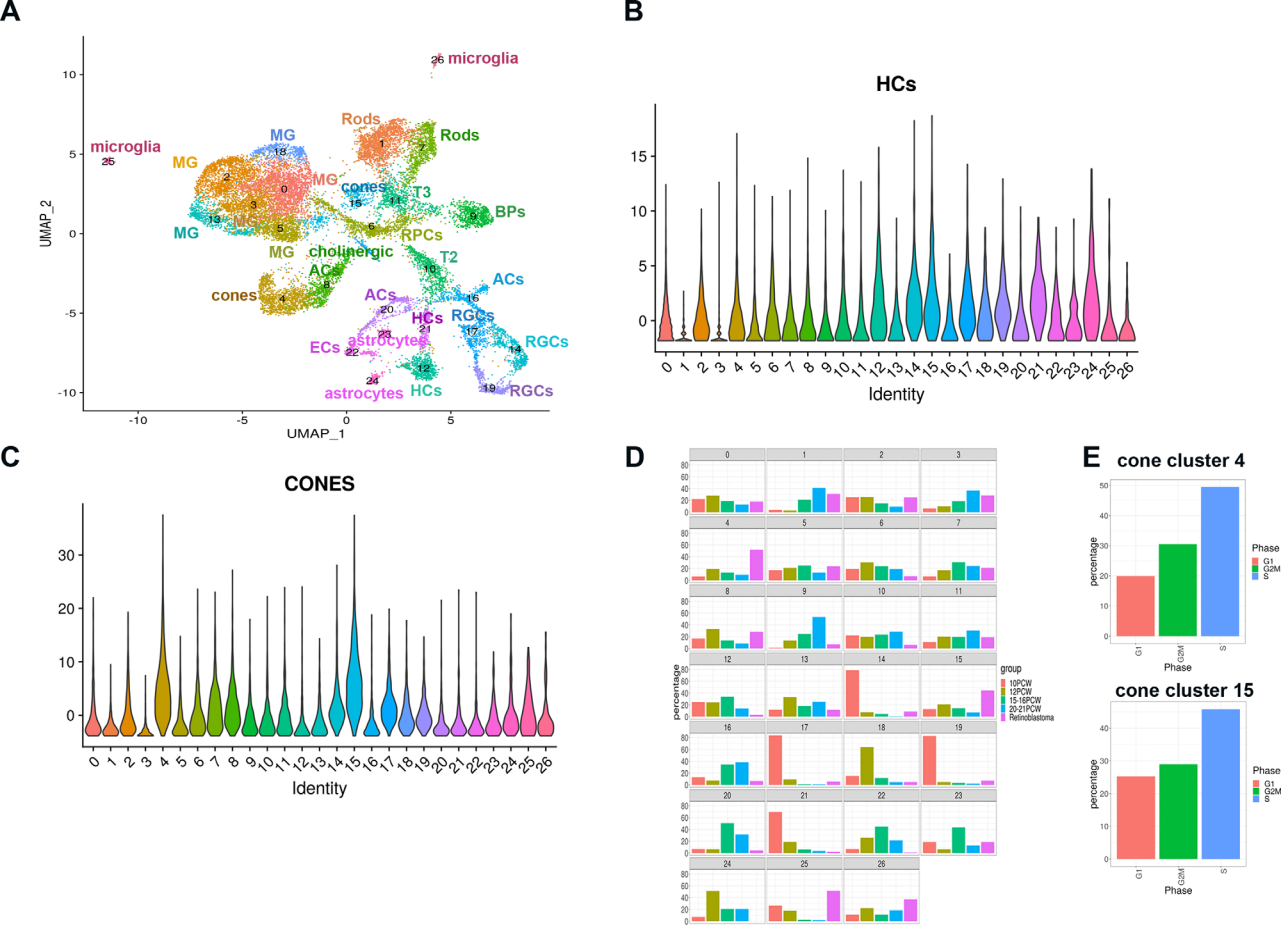
Integrated scATAC-Seq analysis of Rb tumors and human embryonic/fetal retina from 10-21 post-conception weeks (PCW). (**A)** Integrated Uniform Manifold Approximation and Projection showing the presence of 26 cell clusters; (**B, C**) Violin plots showing the expression of horizontal cell (B) and cone markers across the 26 cell clusters identified by scATAC-Seq; (**D**) Comparative analysis showing the enrichment of cone clusters 4 and 15 in the Rb tumor samples versus human embryonic and fetal retina from 10-21 PCW; (**E**) Cell cycle distribution of cone clusters 4 and 15. ACs, amacrine cells; BPs, bipolar cells; HCs, horizontal cells; ECs, endothelial cells; MG, Muller Glia cells; RGCs, retinal ganglion cells. T2 and T3 represent the transitional progenitor populations.

After quality control, doublet cell exclusion and data integration 18,000 cells were obtained from all samples. Twenty-six clusters were identified in a combined dataset created from fetal samples (10PCW – 21PCW) and Retinoblastoma samples (Fig. 3A) and a list of markers with differential accessible chromatin peaks was drawn up (Supplementary Table S3) for all clusters except clusters 2, 3, and 15 where differentially accessible chromatin peaks could not be determined. To identify the clusters, we relied on chromatin accessibility peaks of widely used retinal cell type specific markers (for example, *ONECUT1* and *ONECUT2* for horizontal cells; *GNAT2, RXRG* for cones, etc.^20^^,^^21^; Figs. 3B, 3C). This analysis resulted in identification of all retinal cell types including the transitional progenitor populations T2, T3, recently identified in another scRNA-Seq study of developing human retina.^20^ For clusters 2, 3, and 15, where differentially accessible chromatin peaks could not be identified, we relied on violin plot expression of retinal markers obtained from the scRNA-Seq analysis and comparative differential expression of accessible chromatin peaks to dissect these clusters identity in finer detail (data not shown). This resulted in identification of clusters 2 and 3 as Müller Glia cell clusters (data not shown) and Cluster 15 as cone cell cluster (Fig. 3C). This comparative analysis also indicated that clusters 4, 15, and 25 to 26 (microglia) were much more enriched in Rb tumors when compared to embryonic and fetal human retina samples (Fig. 3D), corroborating the scRNA-seq analysis. The enrichment of microglia is not surprising as the latter have been shown to invade Rb tumors in multiple publications.^23^^,^^28^ Cell cycle analysis indicated that both cone clusters 4 and 15 enriched in Rb tumors were characterized by cells predominantly in G2/M and S phase of the cell cycle (Fig. 3E).

**Figure 4.**
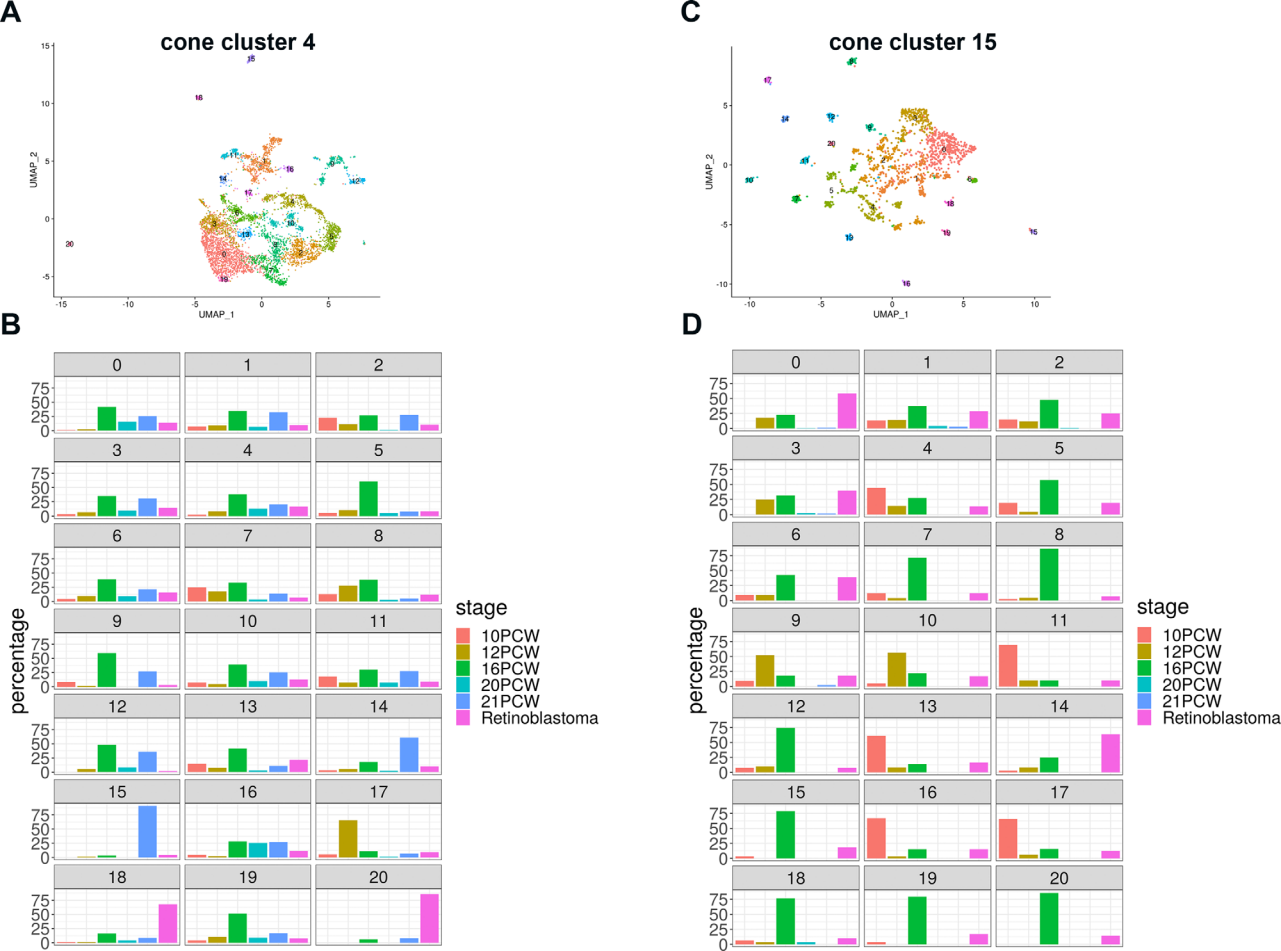
Identification of Rb specific cone subclusters. (**A, B**) Integrated Uniform Manifold Approximation and Projections of cone clusters 4 (**A**) and 15 (**C**) of Rb tumors and human embryonic/fetal retina samples; (**B**) Enrichments of cone cluster 4 subclusters 18 and 20 in Rb tumors; (**D**) Enrichment of cone cluster 15 subclusters 0 and 14 in Rb tumors.

Given the enrichment of cone clusters 4 and 15 in Rb tumor samples, we went on to do further subclustering with the aim of identifying pathways and upstream regulators that were specifically enriched in Rb cones. To facilitate this, we selected cells in cone clusters 4 from fetal and embryonic retina samples and combined these with Rb samples as described above (Fig. 4A), resulting in generation of subclusters, which were further analyzed for enrichment in Rb tumor samples using cell percentage per stage function (Fig. 4B). The same analysis was performed for the cone cluster 15 (Figs. 4C, 4D), leading to identification of subclusters 18 and 20 from cone cluster 4 and subclusters 0 and 14 from cone cluster 15 as being highly enriched in Rb tumor samples. Differential accessibility analysis was then used to test for significant differences in open regions between these Rb enriched-and the rest of cone subclusters (Supplementary Table S4). No differentially accessible peaks were found for subclusters 0 and 14 from cone cluster 15, indicating that although these cone subclusters were enriched in the Rb tumors, in terms of chromatin accessibility, they were similar to cone precursors arising during early retinal development. For this reason, pathway and regulator analysis was performed only for the cone cluster 4 subclusters 18 and 20. Together these data suggest the presence of two Rb tumor specific cone subclusters.

### The Rb Cone Enriched Subclusters are Characterized by Activation of Different Signaling Pathways, Culminating in Escape from Cell Cycle Arrest and Cell Death

**Figure 5.**
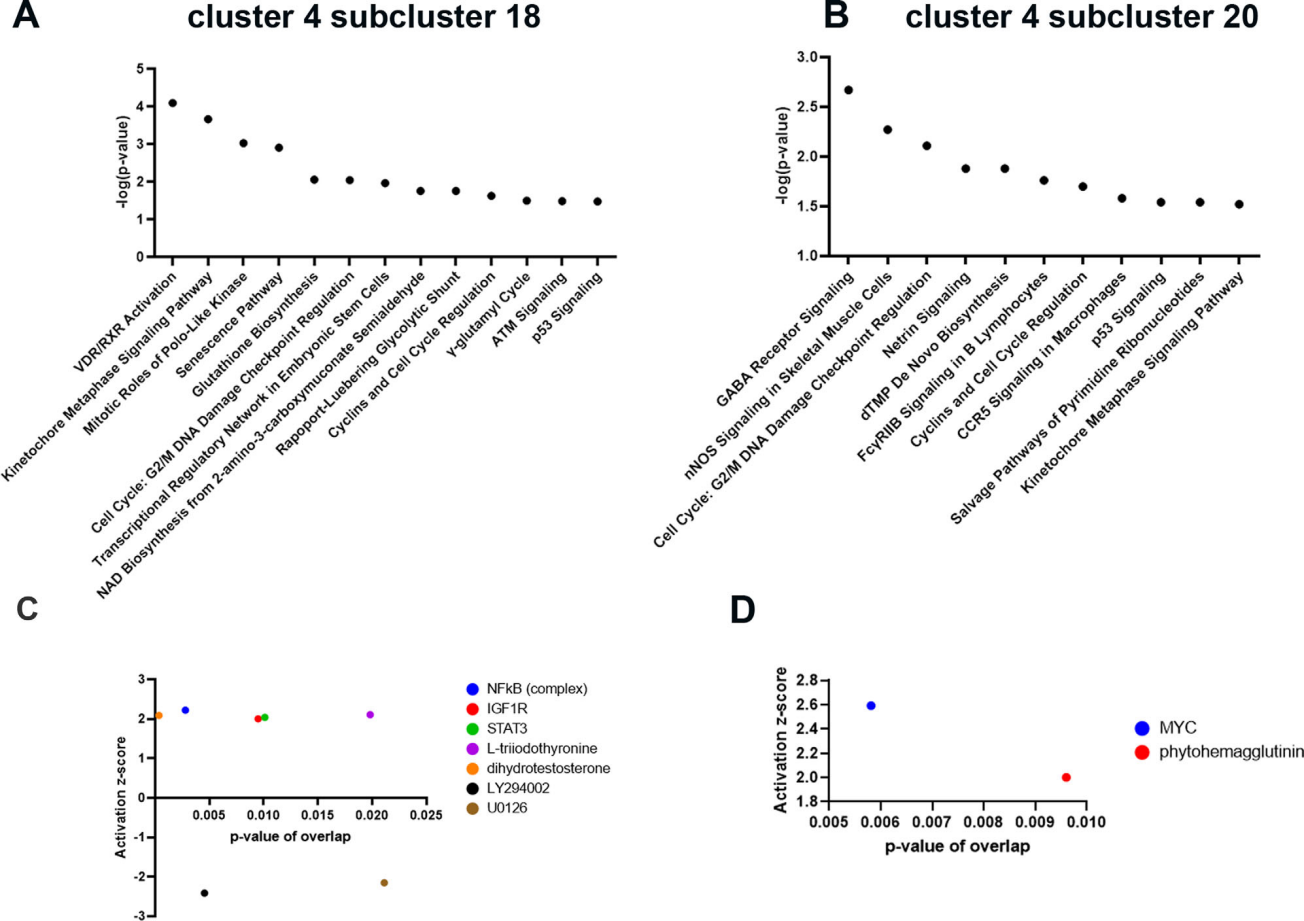
Rb specific cone subsets are characterized by activation of different signaling pathways and upstream regulators. (**A, B**) Top enriched signaling pathways in cone cluster 4, subcluster 18 (**A**) and 20 (**B**) identified by IPA analysis; (**C, D**) Predicted upstream regulators in cone cluster 4, subclusters 18 (**C**) and 20 (**D**).

**Figure 6.**
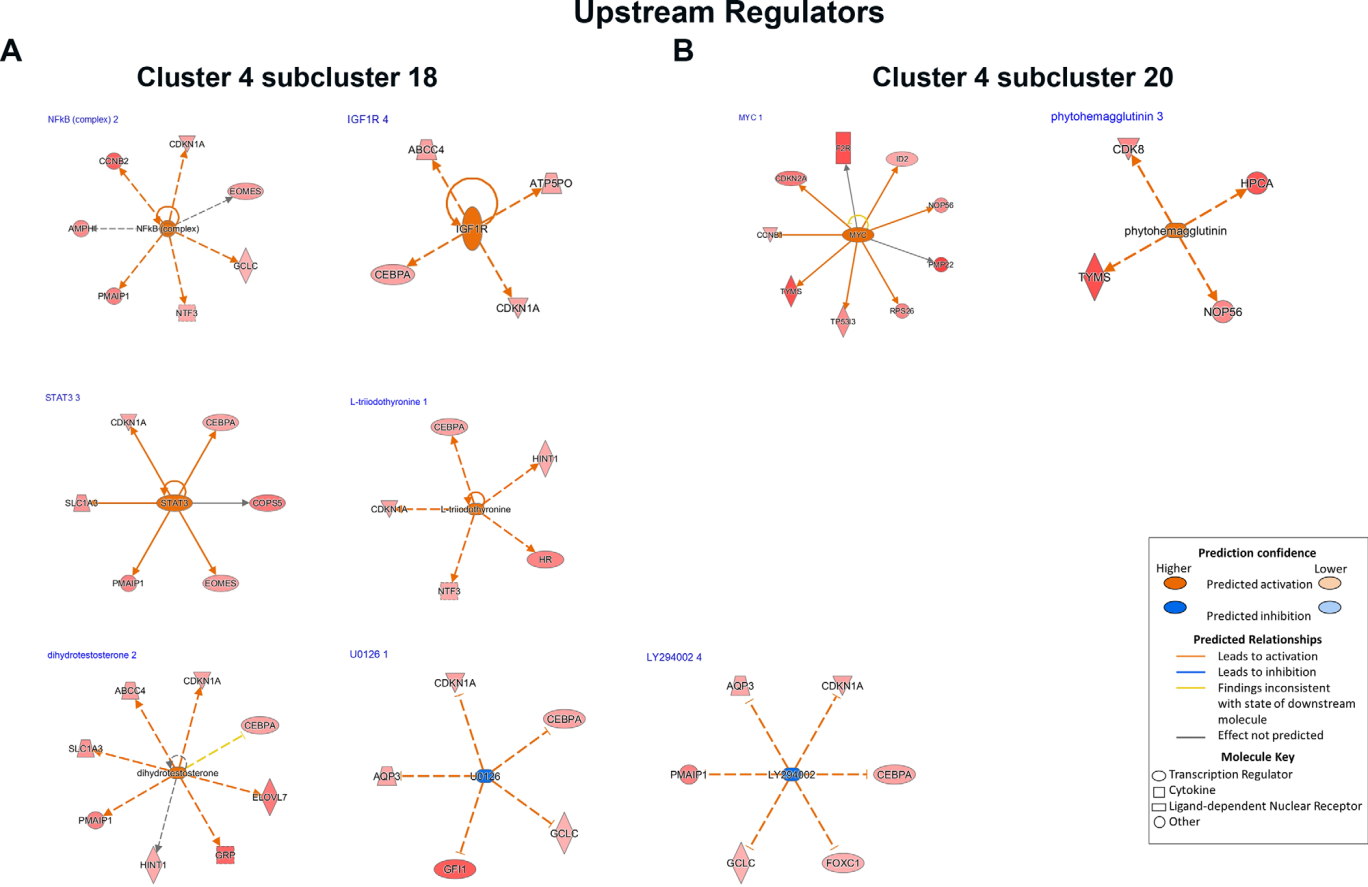
Network analysis of predicted regulators of cone cluster 4, subclusters 18 (**A**) and 20 (**B**). The DA peaks from subclusters 18 and 20 were identified and those were linked to promoters using the Cellranger ATAC pipeline. The JEME database was used to predict peaks in enhancer regions and the JASPAR 2020 database was used to look for the presence of motifs in differentially accessible peaks. The differentially accessible peaks were analyzed in Qiagen IPA. The lists of promoters were used to predict signaling pathways and upstream regulators, whereas motif data were overlaid onto the prediction to look for consensus in the predictions.

To gain insights into upstream regulators and signaling pathways operating in each of the Rb cone enriched subclusters, the differentially accessible peaks identified in subclusters 18 and 20 of cone cluster 4 were linked to promoters using annotation from the Cellranger ATAC pipeline (Supplementary Table S4). The JEME database^17^ was used to predict peaks in enhancer regions and the JASPAR 2020 database^18^ was used to look for the presence of motifs in differentially accessible peaks (Table S4). The differentially accessible peaks were analyzed in Qiagen IPA. The lists of promoters were used to predict signaling pathways and upstream regulators, whereas motif data were overlaid onto the prediction to look for consensus in the predictions (Supplementary Table S5). This analysis indicated activation of specific pathways and upstream regulators for each of the two cone subclusters, characterized by activation of different signaling pathways and upstream regulators following RB1 inactivation (Figs. 5, 6). For example, cone subcluster 20 was associated with activation of upstream regulator *MYC*. *MYC* has been shown to coordinate proliferation of retinal progenitor cells during early retinal development^29^ in cooperation with *MYCN* and its activation in the this Rb specific cone subcluster is likely to lead to cone proliferation, resulting in oncogenic stress and increased *CDKN2A* (Fig. 6B). *CDKN2A* encodes several proteins with two most well studied being the p16(INK4A) and p14(ARF) proteins. The p16(INK4A) binds to CDK4 and CDK6 and blocks their ability to stimulate cell cycle progression. Published findings indicate that p16(INK4A) is upregulated in human retinomas, in response to genomic instability induced by loss of RB1, most likely leading to blocking of cell cycle through RB1 related family members (p130 or p107).^30^ The p14(ARF) protein protects p53 from being broken down via MDM2 or MDM4 degradation.^31^ Nonetheless, MDM2 and MDM4 are both expressed in all Rb cone clusters (Supplementary Fig. S5), likely impeding an ARF-mediated response and further promoting cone proliferation and survival in Rb tumors.^23^ This could be due to high expression of RXRG and THRB, both shown to activate MDM2 and MDM4 expression of lack of detectable p14 protein reported in human RB tumors.^32^ Hence, MDM2 and MDM4 inhibition strategies to ensure p53 mediated apoptosis could be promising for treatment of Rb tumors. Indeed, such approaches have been tested using a specific inhibitor of the MDM2-p53 axis (Nutlin-3a), which has been shown to induce p53-mediated cell death in retinoblastoma cell lines^33^ and xenograft models of retinoblastoma alone or in combination with systemic Topotecan.^34^ Alternatively, targeting of the MYC oncoprotein via a MYC/MAX complex antagonist can be tested to prevent cone proliferation in the first place.^24^^,^^35^

A detailed analysis of cone cluster 4 subcluster 20 also revealed the GABA (γ-aminobutyric acid) receptor signaling as the top enriched pathway (Fig. 5B). GABA is the main inhibitory neurotransmitter in the retina, acting through ionotropic GABA_A_ and GABA_C_ receptors and the metabotropic GABA_B_ receptor.^36^ Various studies in the field have shown that in most cases, GABA stimulates cancer cell proliferation through the GABA_A_ receptor pathway and inhibits cancer cell growth through the GABA_B_ receptor. Earlier studies in human Y-79 retinoblastoma cell line have shown that GABA uptake and binding were absent from these cells.^37^ Further detailed molecular and biochemical studies have shown subsequently that the blockade of monoamine receptors, but not other major neurotransmitter signaling pathways was able to reduce retinoblastoma growth and survival.^38^ Using IPA, the target genes in the GABA signaling pathway, were identified (Supplementary Fig. S7). All three genes, *CACNA2D4, CACNG4* and *KCNQ3* encode calcium and Potassium Voltage-Gated Channels.^39^ There is increasing evidence that both calcium and potassium voltage-gated channels are differentially expressed in cancer cells, and may contribute to cell proliferation, cell cycle regulation, apoptotic resistance, migration and cell invasion through Ca2^+^ or K^+^ dependent or independent signaling pathways.^40^ This raises the possibility of repurposing known calcium and potassium channel blockers for treatment of Rb tumors. Proof of principle for this has been obtained by inhibition of Eag1, a voltage-gated potassium channel, via astemizole, resulting in decreased cell proliferation in retinoblastoma cell cultures.^41^ Another potential strategy can be activation of GABA-B receptor signaling using agonists such as Baclofen, a GABA-B receptor agonist, which can lead to activation of G protein coupled receptor signaling, resulting in inhibition of the calcium and Potassium Voltage-Gated Channels^39^ (Supplementary Fig. S7).

**Figure 7.**
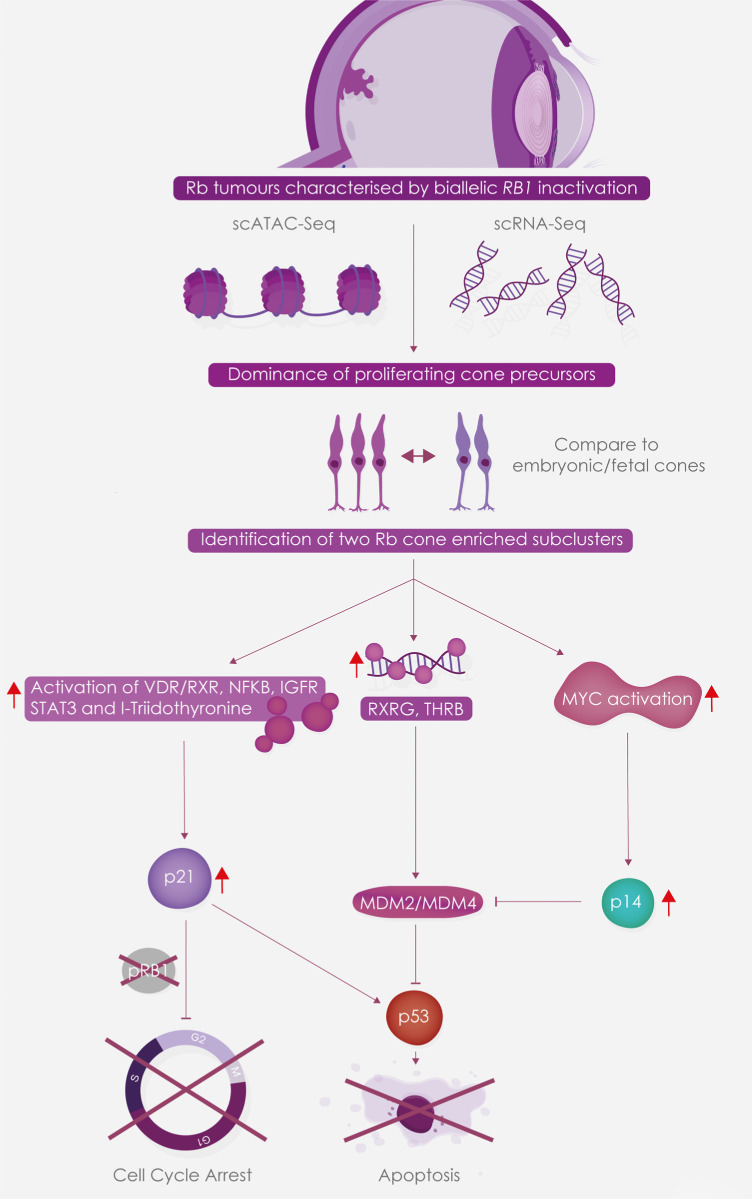
Schematic presentation showing the identification of Rb-enriched cone subclusters associated with activation of signaling pathways and upstream regulators, which culminate in cone precursor escape from cell cycle arrest or apoptosis.

The top signaling pathway identified in Rb cone cluster 4 subcluster 18, was VDR/RXR activation. RXR is a member of the retinoid X receptor family involved in mediating the anti-proliferative effects of retinoic acid (RA). RXR forms heterodimers with RA, thyroid hormone of vitamin D, increasing DNA binding and transcriptional function on their response elements of target genes. Proteins involved in RXR signaling have been reported previously in invasive retinoblastoma tumors; however, these have been linked to formation of a different heterodimer, namely LXR/RXR.^42^^,^^43^ Ligands that can activate the VDR/RXR such as 9-cis RA and vitamin D analogues have been shown to inhibit cell proliferation in a retinoblastoma cell line and attenuate tumor growth in a xenograft model of human Rb respectively.^44^^,^^45^ Importantly, treatment with vitamin D analogues did not interfere with cell proliferation but caused an increase in apoptosis and expression of cyclin dependent kinase (CDK) inhibitor, CDKN1A, also known as p21. IPA analysis also identified upregulation of p21 is a downstream event of VDR/RXR activation in cone cluster 14, subcluster 18 (Supplementary Fig. S8). The primary function of p21 is to inhibit CDKs, maintaining pRb in a hypo-phosphorylated state and causing a G1 cell cycle arrest. However, when both alleles of *RB1* gene are inactivated as in the case of Rb tumors, this G1 cell cycle arrest cannot be imposed. Activation of upstream regulators such as NFKB complex, IGF1R, L-triiodothyronine and STAT3 were also identified in this subcluster. Importantly, a common target gene activated by these upstream regulators (Fig. 6A) was p21, leading us to conclude that p21 activation is a key event in this Rb cone enriched subcluster. In the absence of pRb, p21 is unable to ensue cell cycle arrest, leading to activation of p53, whose degradation is controlled by MDM2 and MDM4 proteins. Interestingly, MDM2 expression has been shown to be regulated by the cone specific RXRγ transcription factor and the cone-specific thyroid hormone receptor THRB, both of which are highly expressed in Rb proliferating cones. Expression of MDM2 and MDM4 in cone clusters is likely to lead to the degradation of p53, which is now unable to execute cell death, leading to cone precursors to escape both cell cycle arrest and cell death (Fig. 7). Hence, targeting either the VDR/RXR or upstream regulators of this subclusters will not be effective, unless MDM mediated degradation of p53 can be resolved, using some of the strategies highlighted above for subcluster 20 (e.g., via MDM2 or MDM4 inhibitors).

## Discussion

In this study we report the first single cell RNA-and ATAC-Seq analyses of two retinoblastoma tumors, caused by biallelic mutations in the *RB1* gene, revealing the dominant presence of proliferating cone precursors at different stages of cell cycle in the tumors and identifying G2/M cone precursors as the cell of origin for Rb. Our combined scRNA- and ATAC-Seq analyses provide detailed molecular evidence, revealing p53 mediated cell apoptosis to be the most likely compromised final event in both of Rb cone specific subclusters. In one of the subclusters this was initiated by activation of MYC, leading to increased expression of *CDKN2A*, which encodes several proteins, two most well studied being p16(INK4A) and p14(ARF). High expression of p16(INK4A) is observed in retinomas and is thought to lead to cell cycle block via p107 or p130.^30^ In contrast, p14(ARF) degrades MDM2 and through this relieves p53 from MDM2/MDM4 dependent degradation to ensue apoptosis. However, in the proliferating cone precursors, MDM2 expression is stimulated by two cone-specific factors, RXRG and THRB, both of which are highly expressed, leading to p53 degradation and cone precursor escape from cell death. In the second cone-specific subcluster, activation of all upstream regulators, as well as the top signaling pathway, resulted in activation of p21, which under normal circumstances (e.g., functional RB1 protein) should lead to cell cycle arrest. However, with RB1 protein being inactivated in Rb tumors, this cell cycle arrest cannot be imposed and thus p21 is thought to activate p53 expression to promote apoptosis, which is also blocked because of MDM2/MDM4 expression in Rb cone cells, also enabling cone precursors of this subcluster to escape both cell cycle arrest and p53-mediated cell death.

Given the predicted *CDKN2A* activation in one of the subclusters but the inability to perform immunofluorescence assays in tumor samples, we cannot predict whether p16(INK4A), and/or p14(ARF) are upregulated in this subcluster. This also does not allow us to fully establish whether this subcluster represents a retinoma or a different subcluster (clone) within the Rb tumors. Notwithstanding we can investigate other markers which distinguish retinomas from Rb tumors, for example Ki67 or Cyclin B1, both of which are highly expressed in Rb, but not retinoma samples.^30^ Detailed assessment of scATAC-Seq data show an enrichment of in the accessibility of Cyclin B1 promoter and enhancer, as well as Ki67 enhancer in this subcluster, supporting its Rb definition.

The tumor suppressor protein p53 provides protection from malignant transformation in many cell types.^34^ The p53 signaling is intact in Rb tumors and shown to regulate cell death in human Rb.^46^ Moreover, overexpression of MDM4 can silence p53.^47^ Previous studies have shown no detectable levels of MDM2 in retinoblastoma cells; however, our single cell RNA-Seq studies indicate low *MDM2* and high *MDM4* expression in all the Rb cone clusters, corroborating previously published data^32^ indicating overexpression of MDM4 in some of the Rb tumors at both mRNA and protein level. In view of these findings, rescuing of p53-mediated cell death by inactivation of MDM4 and MDM2 is a promising therapeutic approach. To this end, an improved ocular formulation of Nutlin-3a, which acts as a small molecule inhibitor of the MDM2-p53 axis, has been developed and shown to yield significant improvements in outcomes compared to other therapies for the treatment of retinoblastoma^34^ when delivered via subconjunctival injections in mouse models. The first nutlin-3a analog to progress to clinical trials, was RG7112; however, this was discontinued from clinical use because of significant toxicity.^48^ Subsequently other MDM2 inhibitors have been developed, nonetheless the drawback of this approach is their low affinity for MDM4. In view of this, dual MDM2-MDM4 inhibitors have been developed with one already in clinical trial evaluations (ARLN-6924, Aileron Therapeutics) including children with Rb.^49^^,^^50^ This inhibitor has been shown to bind with high affinity to both MDM2 and MDM4 and to robustly activate the p53 pathway in acute myeloid leukaemia.^50^ Although the results of the clinical trial have not been reported yet, data from the single-cell study of human Rb tumors strongly supports its application for the treatment of Rb tumors.

## Conclusions

In summary, our study demonstrates successful application of single cell sequencing analyses for unravelling the heterogeneity and molecular pathogenesis of this childhood malignancy. These analyses are well established and quick to perform, hence the route from discovery to identifying potential treatment can be significantly shortened to enable their testing in patient specific disease models and/or xenograft models of Rb. While we recognize that the limitation of this study is the small sample size, this proof-of-principle study paves the way for introducing single cell analyses as a robust method for understanding the heterogeneity at the molecular and cellular level and opens new avenues toward efficacious patient-tailored treatments.

## Supplementary Material

Supplement 1

Supplement 2

Supplement 3

Supplement 4

Supplement 5

Supplement 6

Supplement 7

Supplement 8

Supplement 9

Supplement 10

Supplement 11

Supplement 12

Supplement 13
