# Supplementary material for: Dissecting the Transcriptional and Chromatin Accessibility Heterogeneity of Proliferating Cone Precursors in Human Retinoblastoma Tumors by Single Cell Sequencing—Opening Pathways to New Therapeutic Strategies?
Source: Invest Ophthalmol Vis Sci. 2021 May 17;62(6):18. doi: 10.1167/iovs.62.6.18 (PMC8132003; doi:10.1167/iovs.62.6.18)
Supplement: Supplement 1 [file iovs-62-6-18_s001.pdf]

Figure S1

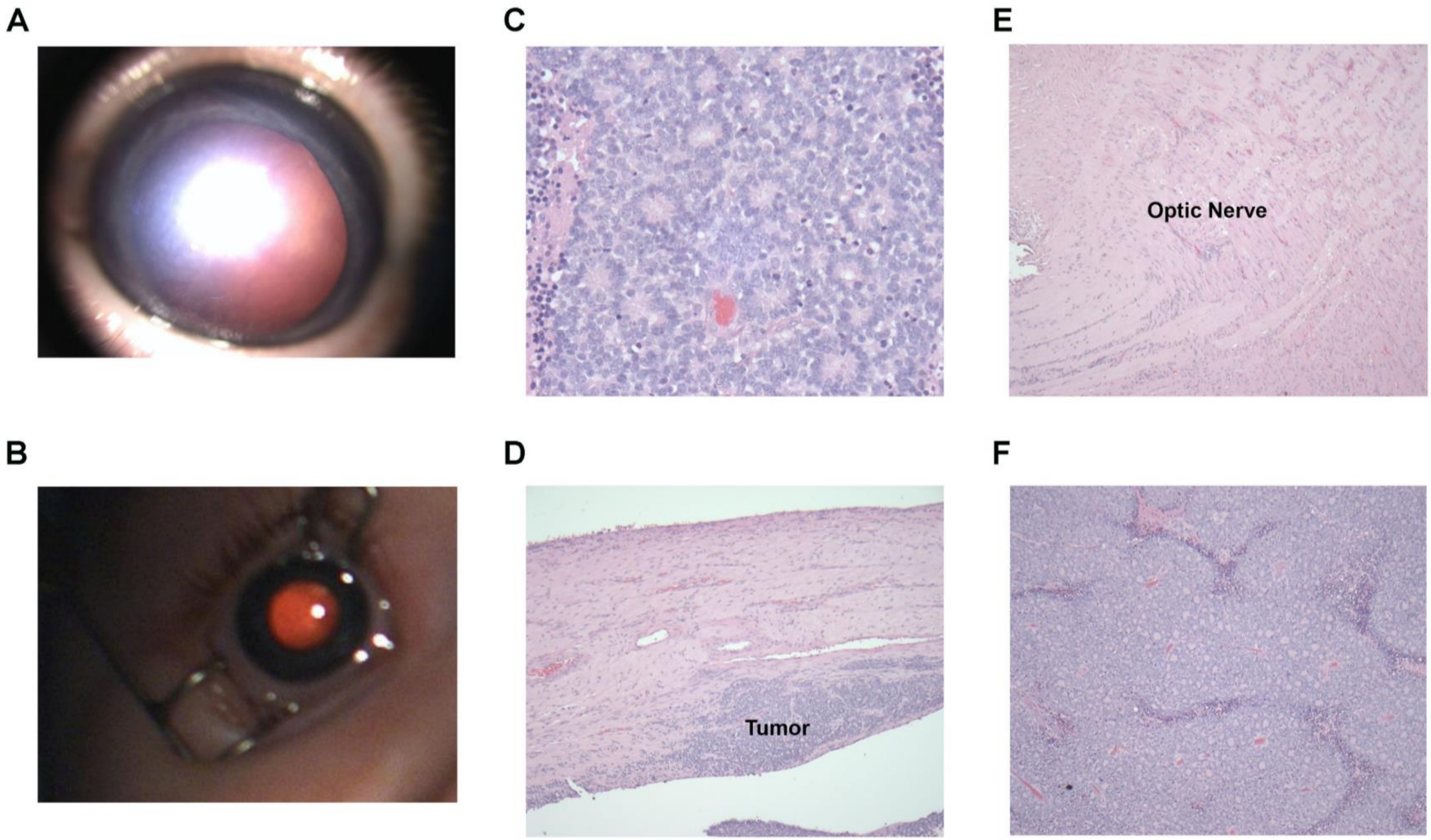

Figure S1: Microphotographs of eye and tumour sections of RB patient 1. A, B) Pictures show enlarged left eye, hazy cornea, and eye full of tumour, with altered iris pattern; **C**) High power Rb with rosettes; **D**) Rb arising from optic nerve head; **E**) Optic nerve with lamina cribrosa showing no tumour invasion; **F**) Well differentiated Rb with numerous rosettes.
