# Supplementary material for: Dissecting the Transcriptional and Chromatin Accessibility Heterogeneity of Proliferating Cone Precursors in Human Retinoblastoma Tumors by Single Cell Sequencing—Opening Pathways to New Therapeutic Strategies?
Source: Invest Ophthalmol Vis Sci. 2021 May 17;62(6):18. doi: 10.1167/iovs.62.6.18 (PMC8132003; doi:10.1167/iovs.62.6.18)
Supplement: Supplement 2 [file iovs-62-6-18_s002.pdf]

Figure S2

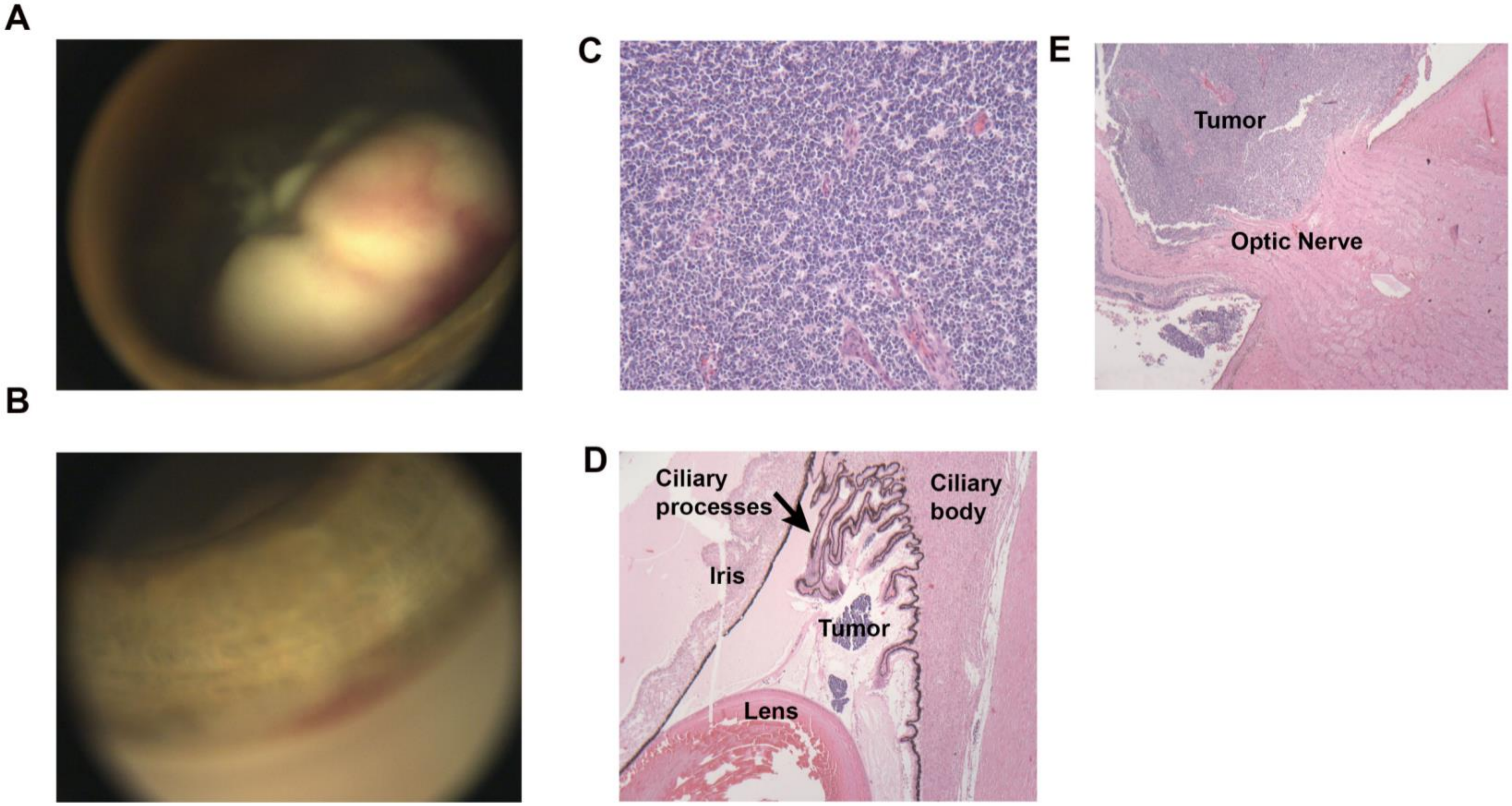

Figure S2: Microphotographs of eye and tumour sections of RB patient 2. A) Picture shows large inferior retinal tumour; **B**) Picture shows blood and tumour in anterior chamber at root of iris; **C**) Viable tumour with Flexner-Wintersteiner rosettes; **D**) Anterior chamber seeding, **E**) Prelaminar optic nerve invasion.
