# Supplementary material for: Dissecting the Transcriptional and Chromatin Accessibility Heterogeneity of Proliferating Cone Precursors in Human Retinoblastoma Tumors by Single Cell Sequencing—Opening Pathways to New Therapeutic Strategies?
Source: Invest Ophthalmol Vis Sci. 2021 May 17;62(6):18. doi: 10.1167/iovs.62.6.18 (PMC8132003; doi:10.1167/iovs.62.6.18)
Supplement: Supplement 3 [file iovs-62-6-18_s003.pdf]

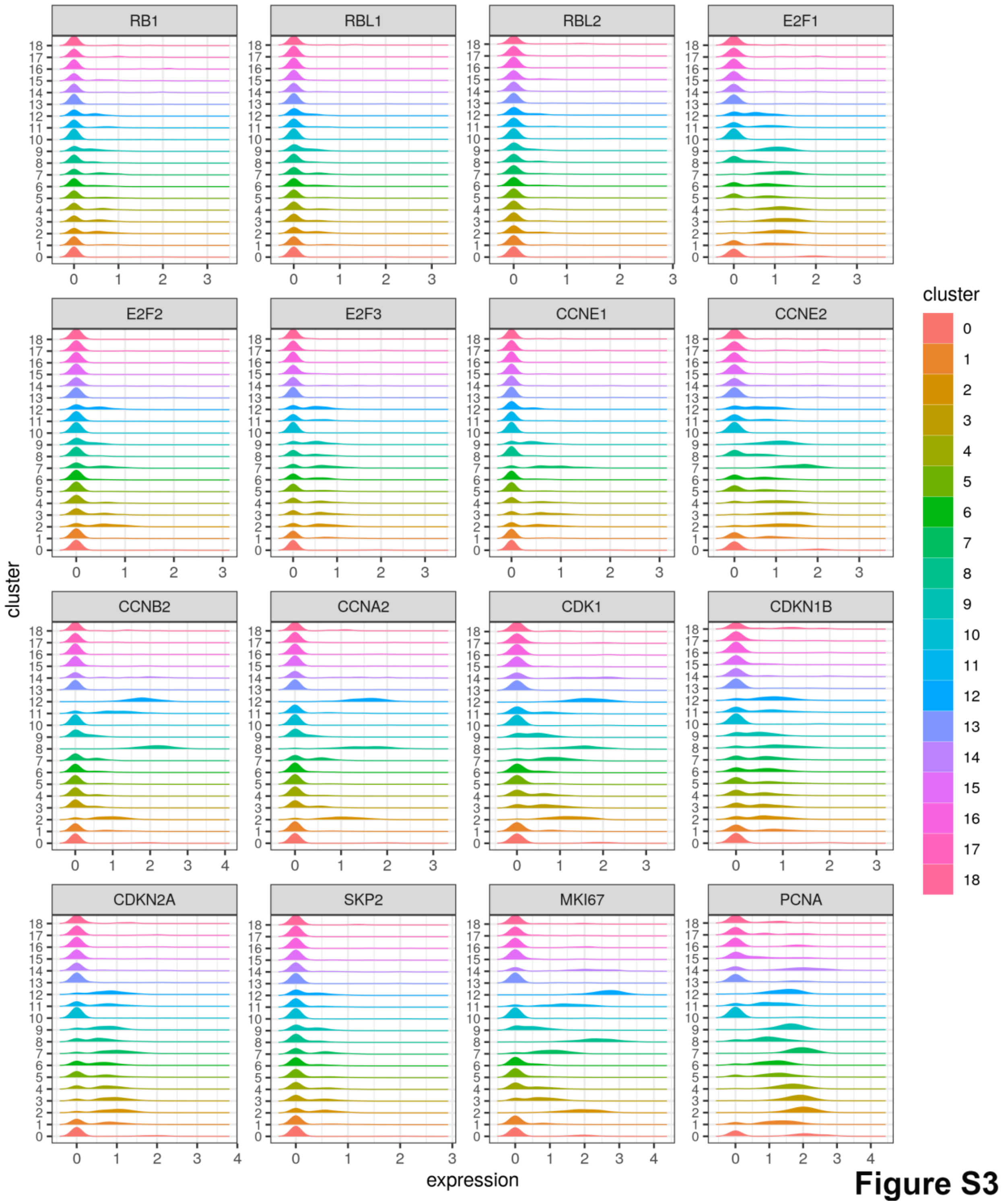

Figure S3: **Composite violin plots showing the expression of *RB1* and its family members *RBL1, RBL2*, and key cell cycle regulators in the 18 cell clusters identified by scRNA-Seq analysis.**
