# Supplementary material for: Dissecting the Transcriptional and Chromatin Accessibility Heterogeneity of Proliferating Cone Precursors in Human Retinoblastoma Tumors by Single Cell Sequencing—Opening Pathways to New Therapeutic Strategies?
Source: Invest Ophthalmol Vis Sci. 2021 May 17;62(6):18. doi: 10.1167/iovs.62.6.18 (PMC8132003; doi:10.1167/iovs.62.6.18)
Supplement: Supplement 4 [file iovs-62-6-18_s004.pdf]

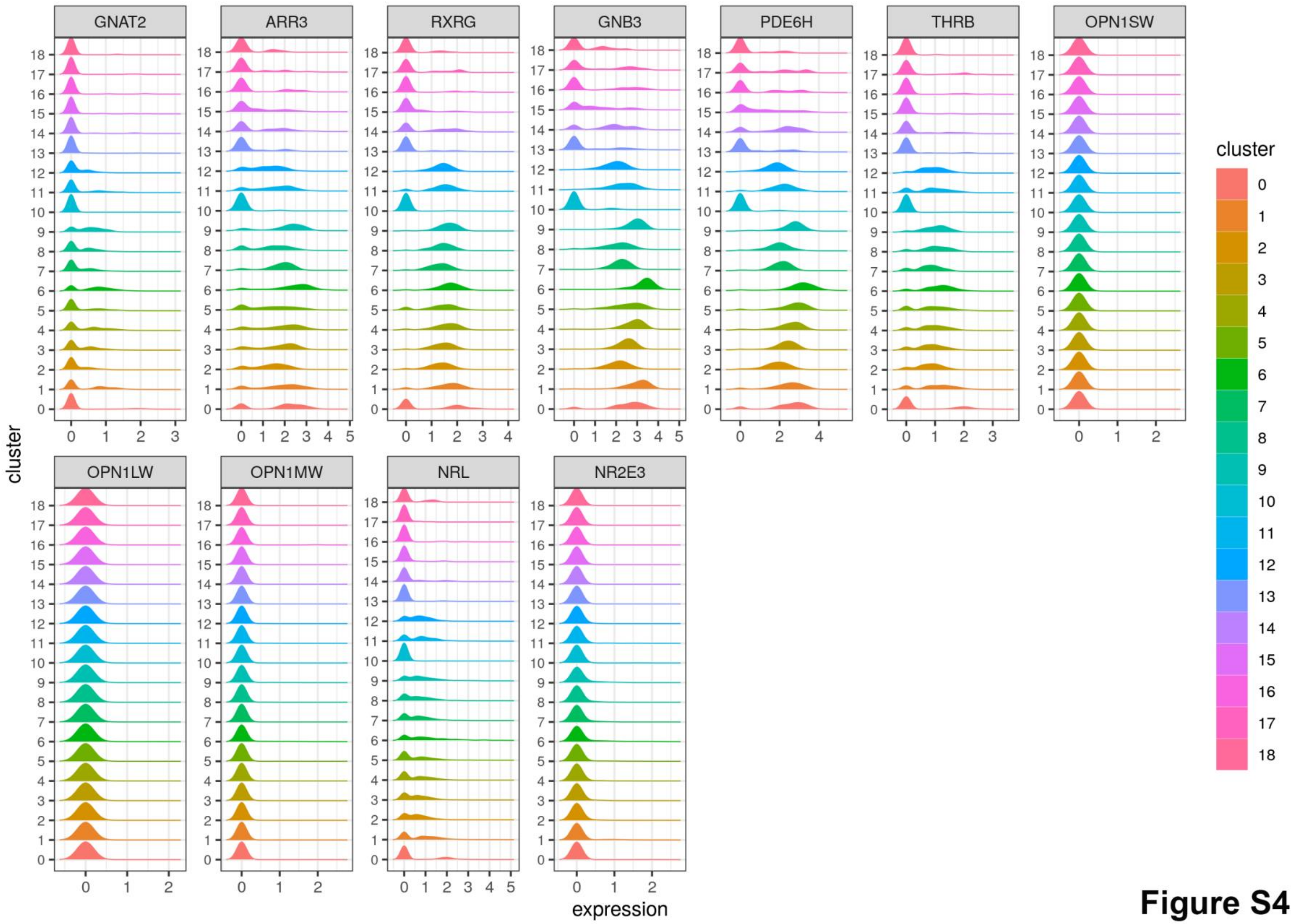
GNAT2
ARR3
RXRG
GNB3
PDE6H
THRB
OPN1SW
OPN1LW
OPN1MW
NRL
NR2E3
cluster
expression
cluster
0
1
2
3
4
5
6
7
8
9
10
11
12
13
14
15
16
17
18

Figure S4

Figure S4: Composite violin plots showing the expression of cone and rod markers in the 18 cell clusters identified by scRNA-Seq analysis.
