# Supplementary material for: Dissecting the Transcriptional and Chromatin Accessibility Heterogeneity of Proliferating Cone Precursors in Human Retinoblastoma Tumors by Single Cell Sequencing—Opening Pathways to New Therapeutic Strategies?
Source: Invest Ophthalmol Vis Sci. 2021 May 17;62(6):18. doi: 10.1167/iovs.62.6.18 (PMC8132003; doi:10.1167/iovs.62.6.18)
Supplement: Supplement 5 [file iovs-62-6-18_s005.pdf]

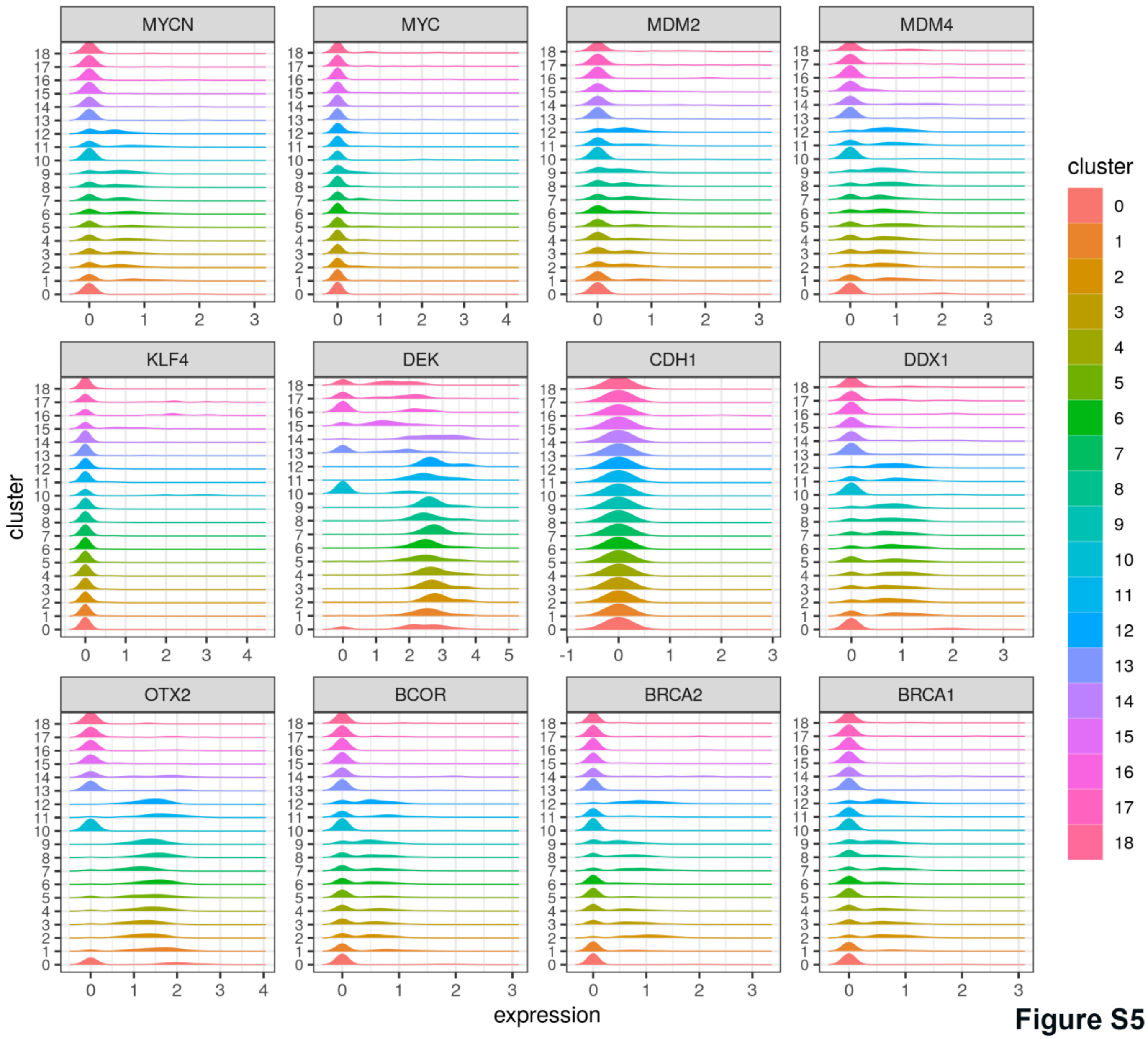
MYCN
MYC
MDM2
MDM4
KLF4
DEK
CDH1
DDX1
OTX2
BCOR
BRCA2
BRCA1
cluster
expression

Figure S5

Figure S5: Composite violin plots showing the expression of key genes implicated in Rb in the 18 cell clusters identified by scRNA-Seq analysis.
