# Supplementary material for: Dissecting the Transcriptional and Chromatin Accessibility Heterogeneity of Proliferating Cone Precursors in Human Retinoblastoma Tumors by Single Cell Sequencing—Opening Pathways to New Therapeutic Strategies?
Source: Invest Ophthalmol Vis Sci. 2021 May 17;62(6):18. doi: 10.1167/iovs.62.6.18 (PMC8132003; doi:10.1167/iovs.62.6.18)
Supplement: Supplement 6 [file iovs-62-6-18_s006.pdf]

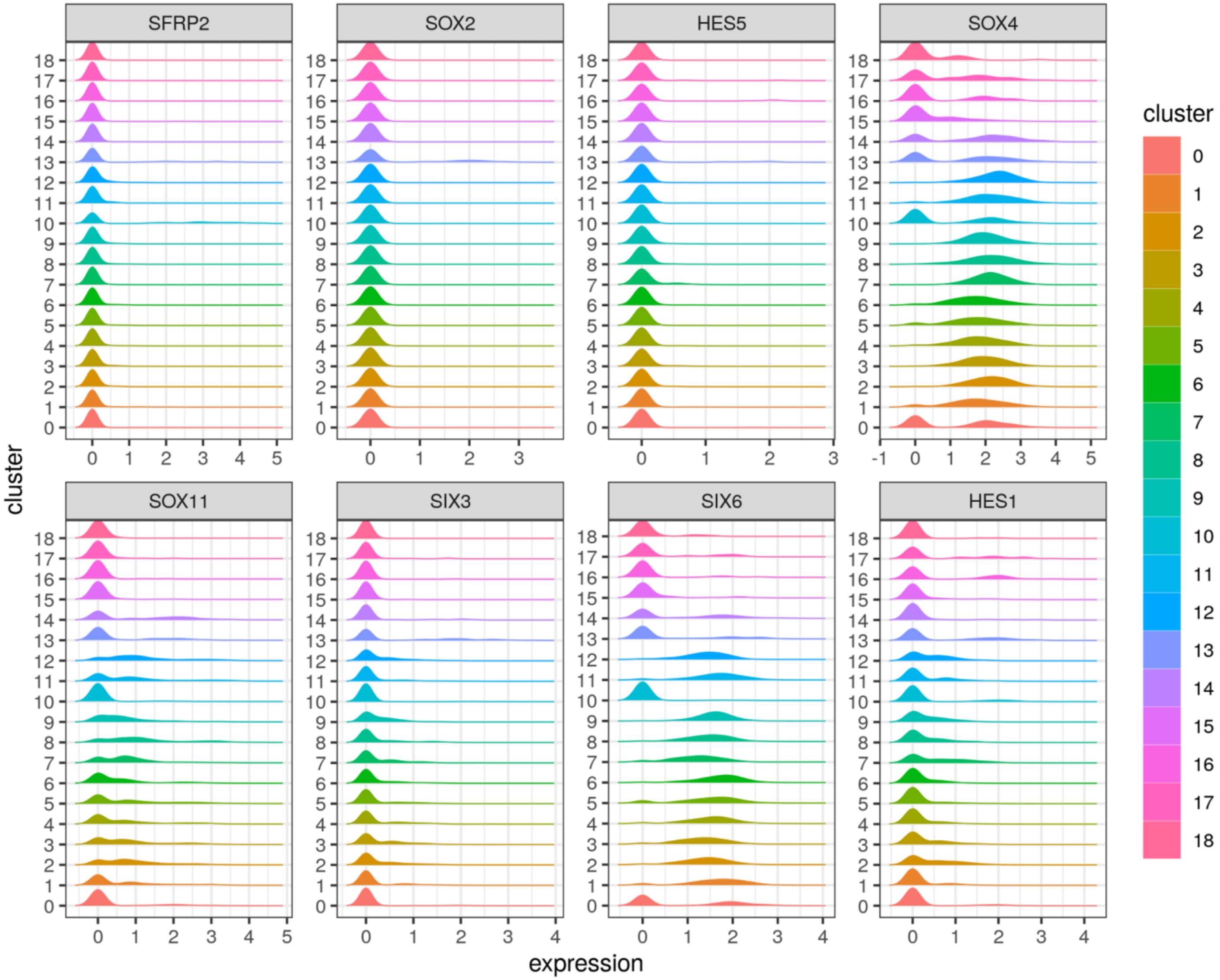
SFRP2
SOX2
HES5
SOX4
SOX11
SIX3
SIX6
HES1
cluster
expression
cluster
0
1
2
3
4
5
6
7
8
9
10
11
12
13
14
15
16
17
18

Figure S6

Figure S6: Composite violin plots showing the expression of retinal progenitor markers in the 18 cell clusters identified by scRNA-Seq analysis.
