# Supplementary material for: Dissecting the Transcriptional and Chromatin Accessibility Heterogeneity of Proliferating Cone Precursors in Human Retinoblastoma Tumors by Single Cell Sequencing—Opening Pathways to New Therapeutic Strategies?
Source: Invest Ophthalmol Vis Sci. 2021 May 17;62(6):18. doi: 10.1167/iovs.62.6.18 (PMC8132003; doi:10.1167/iovs.62.6.18)
Supplement: Supplement 8 [file iovs-62-6-18_s008.pdf]

VDR/RXR activation

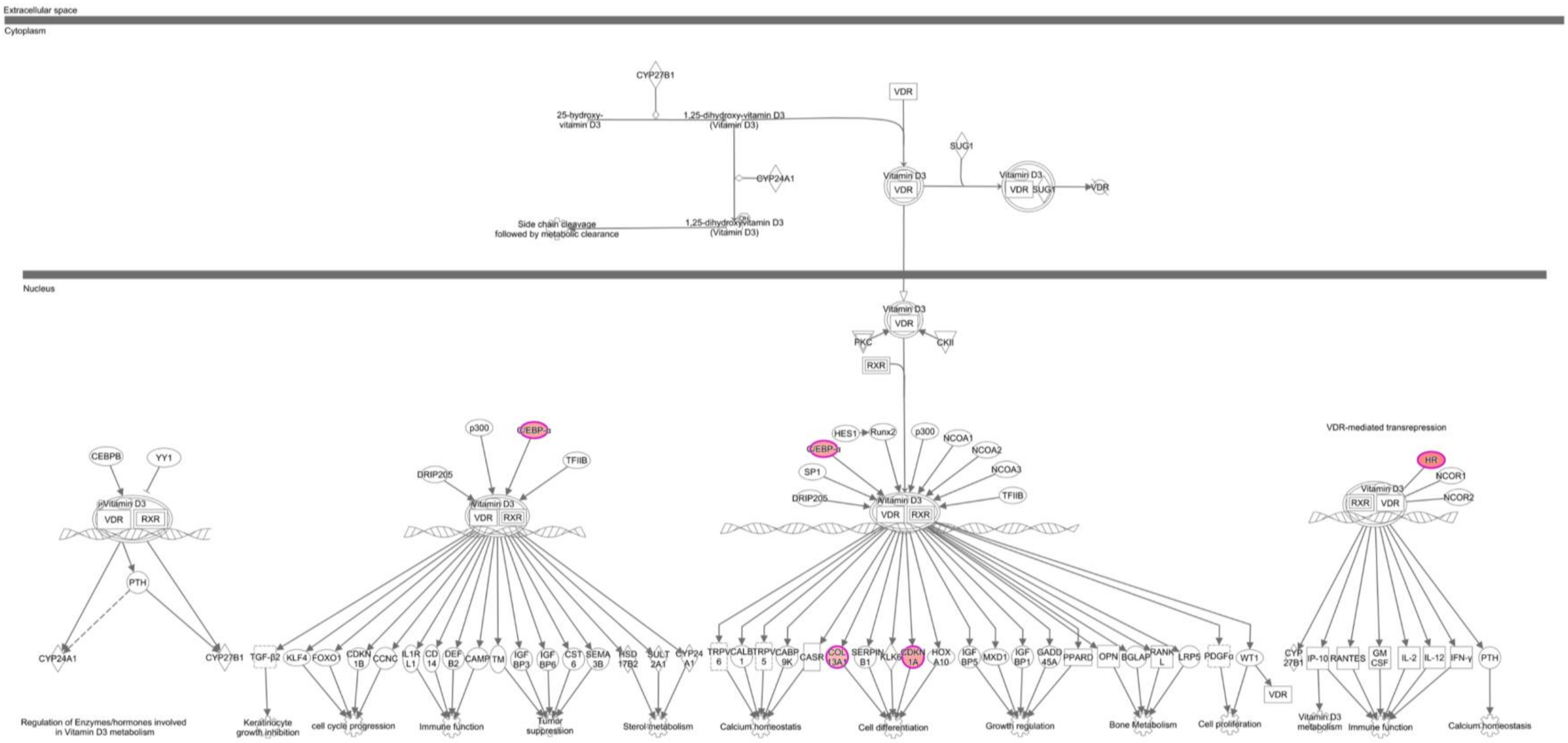

Figure S8

Figure S8: Schematic presentation of VDR/RXR signalling generated by IPA. The target genes identified in the cone enriched Rb subcluster 18 are shown in pink coloured circles.
